# Association of Hearing Loss and Cognitive Decline in the ACTIVE Longitudinal Study

**DOI:** 10.1093/geroni/igaf122.1581

**Published:** 2025-12-31

**Authors:** Nathalie Gider, Caitlin Northcutt, Cynthia Felix, George Rebok, Karlene Ball, Michael Crowe, Tyler Bell

**Affiliations:** University of California, San Diego, San Diego, California, United States; University of Kentucky, Lexington, Kentucky, United States; University of Pittsburgh, Pittsburgh, Pennsylvania, United States; Johns Hopkins University, Baltimore, Maryland, United States; The University of Alabama at Birmingham, Birmingham, Alabama, United States; The University of Alabama at Birmingham, Birmingham, Alabama, United States; University of California, San Diego, San Diego, California, United States

## Abstract

Hearing loss has been identified as a potential risk factor for dementia, but findings remain inconsistent due to methodological limitations, such as failure to account for hearing aid use and comorbidities like depressive symptoms. This study examined the relationship between hearing loss and cognitive decline using data from the Advanced Cognitive Training for Independent and Vital Elderly (ACTIVE) Study (n = 2,802; Mean age=73.63, SD = 5.91; 52.9% women; Mean education=13.35, SD = 2.66). Linear mixed models assessed whether baseline hearing loss predicted changes in Mini-Mental State Examination (MMSE) scores, processing speed (Useful Field of View), reasoning (Letter Series, Number Series, Letter Sets), and memory (Hopkins Verbal Learning Test, Rey Auditory Verbal Learning Test, Rivermead Behavioral Memory Test - Paragraph Recall) over 12 years, adjusting for age, sex, education, race, depressive symptoms, and intervention effects. At baseline, 48.8% of participants (n = 1,205) reported hearing loss, with only 23.2% (n = 280) using hearing aids. Over time, individuals declined in memory (b=-.043, p<.001). Those with hearing loss had worse baseline memory (b=-.256, p<.001) and experienced faster memory decline (b=-.013, p<.001). However, hearing loss was not associated with differences in reasoning or processing speed at baseline or over time (ps>.05), and hearing aid use did not moderate these associations (ps>.05). These findings suggest that hearing loss, regardless of hearing aid use, is linked to worsening memory but not other cognitive domains. Preventing hearing loss may be critical for preserving memory and reducing dementia risk. Addressing hearing loss in aging populations could enhance daily functioning and quality of life.

